# Efficacy of school-based interventions for mental health problems in children and adolescents in low and middle-income countries: A systematic review and meta-analysis

**DOI:** 10.3389/fpsyt.2022.1012257

**Published:** 2023-01-06

**Authors:** Antonio Jose Grande, Mauricio Scopel Hoffmann, Sara Evans-Lacko, Carolina Ziebold, Claudio Torres de Miranda, David Mcdaid, Cristiane Tomasi, Wagner Silva Ribeiro

**Affiliations:** ^1^Department of Medicine, Universidade Estadual de Mato Grosso do Sul, Campo Grande, Brazil; ^2^Care Policy and Evaluation Centre, Department of Health Policy, London School of Economics and Political Science, London, United Kingdom; ^3^Department of Neuropsychiatry, Universidade Federal de Santa Maria, Santa Maria, Brazil; ^4^Graduate Program in Psychiatry and Behavioral Sciences, Universidade Federal do Rio Grande do Sul, Porto Alegre, Brazil; ^5^Departamento de Psiquiatria, Universidade Federal de São Paulo, São Paulo, Brazil; ^6^Department of Medicine, Universidade Federal de Alagoas, Maceió, Brazil; ^7^Department of Public Health, Universidade do Extremo Sul Catarinense, Criciúma, Brazil

**Keywords:** school, mental health, systematic reviews, adolescent, intervention

## Abstract

**Background:**

Implementation of interventions to treat child and adolescent mental health problems in schools could help fill the mental health care gap in low- and middle-income countries (LMICs). Most of the evidence available come from systematic reviews on mental health prevention and promotion, and there is less evidence on treatment strategies that can be effectively delivered in schools. The aim of this review was to identify what school-based interventions have been tested to treat children and adolescents in LMICs, and how effective they are.

**Methods:**

We conducted a systematic review including seven electronic databases. The search was carried out in October 2022. We included randomised or non-randomised studies that evaluated school-based interventions for children or adolescents aged 6–18 years living in LMICs and who had, or were at risk of developing, one or more mental health problems.

**Results:**

We found 39 studies with 43 different pairwise comparisons, treatment for attention-deficit and hyperactivity (ADHD), anxiety, depression, and posttraumatic stress disorder (PTSD), Conduct disorder (CD). Pooled SMD were statistically significant and showed that, overall, interventions were superior to comparators for PTSD (SMD = 0.61; 95% CI = 0.37–0.86), not statistically significant for anxiety (SMD = 0.11; 95% CI = −0.13 to 0.36), ADHD (SMD = 0.36; 95% CI = −0.15 to 0.87), and for depression (SMD = 0.80; 95% CI = −0.47 to 2.07). For CD the sample size was very small, so the results are imprecise.

**Conclusion:**

A significant effect was found if we add up all interventions compared to control, suggesting that, overall, interventions delivered in the school environment are effective in reducing mental health problems among children and adolescents.

**Systematic review registration:**

[https://www.crd.york.ac.uk/prospero/display_record.php?RecordID=129376], identifier [CRD42019129376].

## Introduction

The global prevalence of mental health conditions in children and adolescents is estimated to be around 13.4% (1) and half of the adults diagnosed with one had their first episode during childhood and adolescence (2). Moreover, young people are presenting increasing levels of mental health problems related to increasingly stressful environments (3, 4), particularly in low- and middle-income countries (LMICs), where young people are exposed to several vulnerability factors for the development of mental health conditions, such as violence and material deprivation (5).

Most of the world’s population (80%) live in LMICs, and, yet, only 6% of research on mental health come from these countries (6). Despite the likelihood of positive and consistent effects from high-income countries for decision making, there is a shortage of trained mental health professionals according to reports from the World Health Organisation (WHO), mental health systems in LMICs are not properly equipped/resourced to deliver appropriate mental health care. Therefore, a mental health care gap remains in these countries, which could be filled through the implementation of mental health programmes that could be delivered by non-specialist professionals (7, 8).

As schooling is compulsory in most LMICs (9), this brings opportunities for delivering mental health care and support. Schools are as settings where youth spend a significant proportion of their time–from at least 4 h/day reaching a maximum of 8 h/day and a place where they learn and develop (10). Therefore, schools are a key setting where mental health could be effectively treated, as some evidence has suggested (11, 12). Indeed, a few systematic reviews have shown the potential of school-based interventions in preventing the development of mental health problems. However, such interventions are focussed mostly on anxiety and depression with low to middle Standardized mean difference (SMD) (13–15). Another systematic review on effectiveness of mental health promotion interventions for young people found that some interventions had a positive impact on children and adolescents’ externalising and internalising problem scores and in improving social and emotional wellbeing (16). However, other interventions had no effect on anxiety, depression and PTSD symptoms (16). Additionally, there is a significant return on investment, as some estimates show that for each $1 invested in universal school-based interventions aiming at mental health prevention and promotion, there is an expected $24 economic return in 80 years, resulting from savings in further health/mental health care, improved school outcomes, productivity, and better life chances (17).

Nonetheless, evidence on interventions to treat mental health problems in the school setting, particularly in LMICs, is scarce. Such interventions, if proven to be effective, could help fill the mental health care gap in low resources settings. Therefore, the aim of this review was to identify what are and how effective are school-based interventions used to treat children and adolescents in LMICs.

## Methods

### Protocol and registration

The protocol for this systematic review was previously published in Medicine (Baltimore) (18) and is registered in the International Prospective Register of Systematic Reviews (PROSPERO) under the number CRD42019129376 (19).

### Eligibility criteria

This study is part of a broader systematic review that has been carried out to identify effective interventions to treat child and adolescent mental health problems in LMIC (18). However, for this specific review, only studies on school-based interventions were included.

In summary, we carried out a systematic review of studies published in scientific journals and grey literature, considering the following inclusion criteria:

1. Population: children and adolescents aged 6–18 years, school-age child and adolescent, living in LMIC based on criteria of World Bank Country and Lending Groups (20).
2. Intervention: any school-based intervention.
3. Condition: the systematic review included any child and adolescent mental health problems.
4. Outcome: primary outcomes were defined as the improvement of participants’ mental health symptoms. Studies that did not assess primary outcomes were still included if their interventions targeted the following secondary outcomes: hospitalisation, wellbeing, quality of life, physical social, or occupational functioning/impairment.
5. Study design: we included randomised and non-randomised controlled trials.
6. Language: there were no language restrictions.
7. Timeframe: studies published from 2007 to 2022 were included.

### Information resource

Only studies published from 2007 onwards were included because this is the year in which child and adolescent mental health became prominent as a global public health challenge (7). Our research was limited to studies published until October 2022.

### Search

An electronic search was carried out in the following databases: MEDLINE Ovid, EMBASE Ovid, PsycINFO Ovid, CINAHL plus, LILACS (Latin American and Caribbean Health Sciences), BDENF (Brazilian Nursing Database), and IBECS (The Spanish Bibliographic Index of the Health Sciences). We also checked reference lists of all included studies and relevant review articles identified through our search for additional references. We emailed experts in the field about other published and unpublished studies that might be eligible for inclusion. No unpublished data were included in this review.

Details on our search strategy and other relevant methodological aspects of our review can be found in our study protocol, which has been previously published (18).

### Study selection

To ensure reliability between reviewers, we performed a screening team training phase, in which 5% of all references were independently screened by 2 different reviewers. An expert in mental health researcher (WSR) resolved divergences independently and made the final decision when it was necessary. Based on the identification of the main reasons for divergences between reviewers, a meeting was held with the review team to clarify potential doubts and solve any systematic error when screening references.

After divergences in the pilot phase were solved and the screening team was retrained, the remaining 95% of references were equally split among the reviewers to finalise the screening phase. Based on our inclusion and exclusion criteria, reviewers read titles and abstracts and classified references into three categories: “no,” “yes,” and “maybe.” References classified as “no” were excluded. Those classified as “yes” or “maybe” were selected for the full-text screening phase, and were analysed again against inclusion/exclusion criteria after full texts have been obtained and read.

The selection process was documented with a Preferred Reporting Items for Systematic Reviews and Meta-Analysis (PRISMA) flowchart (21).

The web based Covidence (covidence.org) tool will be used to perform the management and screening of references, and data extraction from eligible studies.

### Data collection process

#### Data items

We first extracted relevant data from studies, including key characteristics of studies and parameters of interventions’ efficacy/effectiveness.

##### Study details

Aim, study design, design details, country in which study was conducted, details on location of intervention delivery, target condition, or risk factor (i.e., subthreshold symptoms, experience of child maltreatment).

##### Participants

Sample size (intervention and control groups at baseline and follow-up), sociodemographic characteristics (e.g., age, gender, ethnicity, socioeconomic status).

##### Interventions

Description of intervention including frequency and duration, number of sessions, mode of delivery (e.g., face to face, internet), format (e.g., one to one or group), cost of intervention.

##### Delivery of the intervention

Setting in which intervention was delivered (e.g., school), who delivered the intervention (e.g., medical doctor, nurse, psychologist, teacher, lay health worker, etc.) and whether it was delivered by 1 practitioner or a team of individuals, whether there was intersectoral collaboration (e.g., between health and education or guardianship councils).

##### Comparison groups

Characteristics of and procedures for selection comparison groups (e.g., matching vs. randomisation).

##### Outcomes

Primary outcomes will include reduction of mental health symptoms, or improvement in mental health functioning. Secondary outcomes will include, economic impact, reduction of hospitalisations, or improvement in wellbeing, quality of life, resilience, social, physical, and occupational functioning, including educational outcomes.

Studies with missing data were excluded after two unsuccessful attempts of contacting authors. Then, based on pre- and post-intervention scores we estimated within-group mean differences, as well as between-groups (intervention vs. control) mean differences and pooled standard deviations (SD). Mean differences were, then, divided by pooled SDs to be converted into standardized mean differences (SMD).

#### Data analyses

We started our analytical approach by carrying out a descriptive analysis, in which we reported the number of references that were found and dealt with—from number of references retrieved by our search to the number of studies included in the study—, according to the updated version of the PRISMA guideline for reporting systematic reviews (21). Afterwards, we reported key characteristics of studies included in our review by summarising the frequency and proportions of studies in each category of relevant variables—e.g., country, types of interventions, outcomes etc.

For our pairwise meta-analysis, we grouped individual studies into the following emerged conditions: ADHD, PTSD, anxiety, depression, and conduct disorder, interventions have been tested in the school setting. For all other conditions included in our search strategy, interventions were tested only in clinical settings.

### Risk of bias within individual studies

Two review authors (AG and WR) independently critically appraised the studies, all disagreements were resolved by discussion. We used the Cochrane Collaboration’s risk of bias tool, version 2.0. Six parameters were used to assess included studies: (1) Bias arising from the randomisation process; (2) bias due to deviations from intended intervention; (3) bias due to missing outcome data; (4) bias in measurement of the outcome; (5) bias in selection of the reported result; and (6) overall risk of bias of included studies. Based on these parameters, studies were classified into three categories: low risk of bias; some concerns; and high risk of bias (22).

### Summary measures

For our data synthesis, we performed a random-effect pairwise meta-analysis using Stata’s *metan* command, stratified by conditions. Therefore, we estimated pooled standardised mean differences with 95% confidence intervals for each one of the conditions, and an overall pooled mean difference and 95% confidence interval for the combined effect of all comparisons included in the meta-analysis. This approach also allowed us to estimate *I*^2^ parameters of heterogeneity for each one of the subgroups and for the overall pooled analysis.

## Results

### Key characteristics of included studies

**FIGURE 1:**
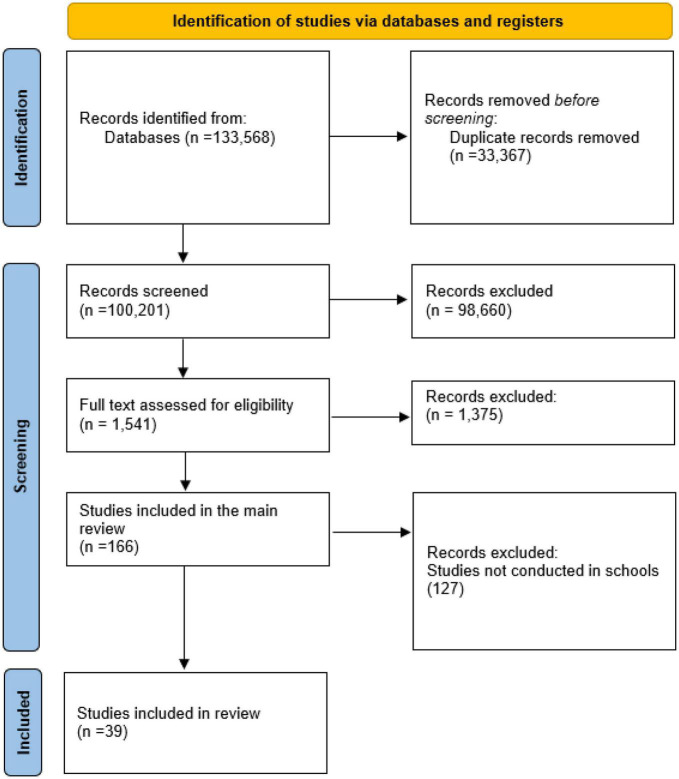
PRISMA flow-chart of systematic review on school-based interventions for children and adolescent in LMIC.

A total of 133,568 references were identified through our search strategy. After the screening of titles and abstracts, and eligibility assessment, 166 studies were in LMIC (Figure 1), 127 were excluded with reason and 39 were on school-based interventions (23–61). We excluded, therefore, 166 studies that were not conducted in schools.

Some of these studies had multiple intervention groups (29, 37), thus a total of 6 interventions were compared in which a total of 9,017 participants were allocated between intervention and control groups.

The included studies were from: Bosnia (34, 39) (*N* = 2–5.13%), Brazil (42, 49) (*N* = 2–5.13%), Burundi (46) (*N* = 1–2.56%), Chile (24, 32) (*N* = 2–5.13%), China (37, 53) (*N* = 2–5.13%), Congo (41) (*N* = 1–2.56%), Kenya (51, 56, 57, 59) (*N* = 4–10.26%) India (45) (*N* = 1–2.56%), Indonesia (48) (*N* = 1–2.56%), Iran (23, 30, 31, 34, 36, 39, 40, 54) (*N* = 8–20.52%), Lebanon (62) (*N* = 1–2.56%), Malaysia (44, 60) (*N* = 2–5.13%), Mexico (61) (*N* = 1–2.56%), Nepal (35) (*N* = 1–2.56%), Nigeria (27, 58) (*N* = 2–5.13%), Palestine (26) (*N* = 1–2.56%), South Africa (55) (*N* = 1–2.56%), Sri Lanka (28, 47) (*N* = 2–5.13%), Turkey (25) (*N* = 1–3.85%), Uganda (50) (*N* = 1–2.56%). Table 1 shows the characteristics of individual included studies.

**TABLE 1:** Characteristics of individual included studies.

References	Country	Funding	Population age range or mean (SD)	Sample	Females	Outcome	Intervention	Comparison	Duration	Follow-up length	Deliverer	SMD (95% CI)	ROB
**ADHD**													
Abadi et al. (23)	Iran	NR	9–12	40	NR	ADHD symptoms	Yoga	No intervention	8 weeks	8 weeks	NR	1.31 (1.01–1.60)	3
Kiani et al. (36)	Iran	NR	13–15	30	30 (100%)	Working memory	Mindfulness meditation	Wait list	8 weeks	8 weeks	Psychologist	2.57 (−0.46 to 5.59)	3
Lan et al. (37)	China	Public	10.9 (1.3)	96	44 (45.83%)	Hyperactivity symptoms	Executive function training	Computerised executive function training	3 months	NR	Researcher	0.74 (−0.16 to 1.64)	1
						Hyperactivity symptoms	Social skills training	Computerised executive function training	3 months	NR	Researcher	−0.09 (−0.67 to 0.49)	1
Pisacco et al. (42)	Brazil	Public	13.1 (1.8)	47	13 (27.65)	ADHD symptoms	Text production + working memory training	Working memory training	3 months	6 weeks	Psychologist	0.47 (0.39–0.55)	2
Haack et al. (61)	Mexico	Public	7.4 (1.36)	58	43 (74.13%)	ADHD symptoms	Comprehensive psychosocial	No intervention	6 weeks	6 weeks	Trained SMHP	−1.04 (−1.59 to −0.49)	3
Lan et al. (53)	China	Public	10.54 (1.16)	96	29 (30.21%)	ADHD symptoms	Group executive function training (GEFT)	Wait list	12 weeks	12 weeks	Psychologist	−0.29 (−0.82 to −0.23)	3
**PTSD**													
Barron et al. (26)	Palestine	Private	11–14	133	60 (45.11%)	PTSD symptoms	CBT-based trauma recovery programme	No intervention	5 weeks	7 weeks	Teacher	Missing	3
Berger and Gelkopf (28)	Sri Lanka	Not reported	9–15	166	79 (47.60%)	PTSD symptoms	ERASE Stress Programme	No intervention	12 weeks	12 weeks	Teacher	0.93 (0.67–1.93)	3
Chen et al. (29)	China	Private	14.5 (0.7)	40	27 (67.50%)	PTSD symptoms	Sort-term CBT	No intervention	6 weeks	3 months	Psychologist	1.27 (−1.69 to 4.24)	3
						PTSD symptoms	General support intervention	No intervention	6 weeks	3 months	No-specialised volunteers	0.42 (−2.04 to 2.88)	3
Hasanovi and Hasanbaši (33)	Bosnia	Not reported	12–15	408	267 (65.44%)	PTSD symptoms	Psychosocial assistance	No intervention	5 weeks	Not reported	Researcher	0.67 (0.34–1.00)	3
Jaberghaderi et al. (34)	Iran	Not reported	8–12	139	69 (49.64%)	PTSD symptoms	CBT	No intervention	12 weeks	14 weeks	Psychologist	1.37 (0.73–2.01)	3
Jordans et al. (35)	Nepal	Private	11–14	325	158 (48.61%)	PTSD symptoms	CBT + cooperative play + expressive exercises	No intervention	5 weeks	Not reported	Researcher	0.01 (−0.09 to 0.10)	3
Layne et al. (38)	Bosnia	Public	13–19	127	82 (64.57%)	PTSD symptoms	Trauma and grief component therapy	Psychoeducation	12 months	16 months	Psychologist	0.49 (−0.08 to 1.05)	3
O’Callaghan and McMullen (41)	Democratic Republic of Congo	Private	14–17	50	21 (42.00%)	PTSD symptoms	Trauma-focussed CBT	Not reported	6 months	6 months	No-specialised health worker	2.48 (1.44–3.53)	1
Qouta et al. (43)	Palestine	Private	10–13	482	NR	PTSD symptoms	Teaching recovery techniques	No intervention	4 weeks	Not reported	Lay counsellors	0.54 (0.42–0.67)	3
Tol et al. (48)	Indonesia	Private	7–15	403	196 (48.63%)	PTSD symptoms	CBT + cooperative play + expressive exercises	No intervention	5 weeks	7 months	No-specialised volunteers	0.45 (0.33–0.58)	3
Tol et al. (47)	Sri Lanka	Private	9–12	399	154 (38.60%)	PTSD symptoms	CBT + cooperative play + expressive exercises	No intervention	5 weeks	4 months	No-specialised volunteers	−0.37 (−0.066 to −0.07)	3
Tol et al. (46)	Burundi	Private	8–17	329	158 (48.02%)	PTSD symptoms	CBT + cooperative play + expressive exercises	No intervention	5 weeks	3 months	No-specialised volunteers	0.06 (−0.33 to 0.45)	3
Getanda and Vostanis (57)	Kenya	Public	14–17 years	54	NI	PTSD symptoms	Writing for recovery (Psycho-social-educational)	Waiting list	3 days	1 week	Facilitator (school counsellor and teacher)	−2.78 (−3.54 to −2.02)	2
Rossouw et al. (55)	South Africa	Public	15.35 (1.46)	63	31 (49.20%)	PTSD symptoms	Prolonged exposure therapy for adolescents	Supportive counselling	14 weeks	24 months	Non-specialised health worker	−1.01 (−1.53 to −0.48)	1
**Anxiety**													
Aydin et al. (25)	Turkey	Not reported	12–14	44	NR	Social anxiety symptoms	CBT	No intervention	13 weeks	13 weeks	Researchers	0.98 (−0.42 to 2.37)	2
Ebesutani et al. (30)	Iran	Not reported	8–11	11	11 (100%)	Anxiety symptoms	Modular CBT	No intervention	10 weeks	10 weeks	Therapist	1.80 (−3.08 to 6.92)	2
Ebrahiminejad et al. (31)	Iran	Not reported	14.5 (4.3)	30	30 (100%)	Social anxiety symptoms	Mindfulness-based cognitive therapy	No intervention	8 weeks	8 weeks	Researchers	0.54 (−1.36 to 2.44)	2
Ab Ghaffar et al. (60)	Malaysia	Public	10–11	461	258 (55.9)	Anxiety symptoms	School program on worry coping skills and self-esteem	Usual school CV	6 weeks	12 weeks	Research assistant	−0.12 (−0.31 to 0.06)	1
Maalouf et al. (62)	Lebanon	Private	12 (0.50)	270	146 (54.10)	Anxiety symptoms	Mental health program	Waiting list	10 weeks	12 weeks	Trained mental health professionals	Missing	3
Osborn et al. (59)	Kenya	Public	15.4 (1.2)	413	268 (64.90)	Anxiety symptoms	Shamiri	Study skills	4 weeks	28 weeks	Layperson (trained)	0.29 (0.07–0.52)	1
Venturo-Conerly et al. (51)	Kenya	Public	16 (1.44)	895	454 (50.72)	Anxiety symptoms	Growth, value, gratitude	Study skills	1 day	2 weeks	Layperson (trained)	−0.04 (−0.23 to 0.15)	1
**Depression**													
Araya et al. (24)	Chile	Private	14.5 (0.9)	2,508	1115 (44.45%)	Depressive symptoms	CBT	No intervention	3 months	12 months	Trained facilitator	0.05 (0.02–0.08)	2
Bella-Awusah et al. (27)	Nigeria	Private	14–17	40	28 (70.00%)	Depressive symptoms	CBT	No intervention	16 weeks	16 weeks	Psychiatrist	1.27 (0.14–2.40)	2
Gaete et al. (32)	Chile	Private	15.9 (0.9)	342	172 (50.29%)	Depressive symptoms	CBT	No intervention	8 weeks	8 weeks	Psychologist	0.08 (−0.17 to 0.32)	2
Neshat-Doost et al. (40)	Iran	Public and private	14.9 (1.9)	23	11 (47.82%)	Depressive symptoms	Memory specific training	No intervention	5 weeks	13 weeks	Psychologist	Missing	2
Saw et al. (44)	Malaysia	Public	16	20	10 (50.00%)	Depressive symptoms	CBT	No intervention	8 weeks	12 weeks	Teacher	2.84 (0.54–5.14)	2
Singhal et al. (45)	India	Public	13–18	120	NR	Depressive symptoms	Coping-skills programme	Interactive psychoeducation	8 weeks	12 weeks	Researchers	4.94 (4.75–5.14)	2
Byansi et al. (50)	Uganda	Public	15.43 (0.90)	1,260	1260 (100%)	Depressive symptoms	Financial literacy + family-based dialogue	Usual school CV	16 weeks	12 months	Assistant researchers	−0.29 (−0.43 to −0.15)	1
Lima et al. (49)	Brazil	Public	13–16	1,296	724 (55.86%)	Depressive symptoms	Doubling PE	No intervention	16 weeks	0	PE teachers	−0.05 (−0.22 to 0.12)	3
Osborn et al. (56)	Kenya	Public	15.75 (1.00)	51	31 (60.78%)	Depressive symptoms	Shamiri	Study skills	4 weeks	0	Layperson (trained)	−0.52 (−1.08 to 0.03)	1
Taghvaienia and Zonobitabar (54)	Iran	Public	16.84 (3.17)	49	49 (100%)	Depressive symptoms	Positive intervention	No intervention	2 months	0	Positive intervention trained coach	−0.61 (−1.19 to −0.04)	1
**Conduct disorder (CD)**													
Kumuyi et al. (58)	Nigeria	None	13–17	16	5 (31.25%)	CD	Combined CBT and SST	No intervention	8 weeks	8 weeks	Researcher	−7.82 (−13.38 to −2.27)	1

ADHD, attention deficit hyperactivity disorder; CBT, cognitive behaviour therapy; ERASE, enhancing resiliency amongst student experiencing stress; NR, not reported; PTSD, post-traumatic stress disorder; ROB, risk of bias; SD, standard deviation; SMD, standard mean difference; 95% CI, 95% confidence interval; 1, low risk of bias; 2, some concerns; 3, high risk of bias.

Interventions evaluated were: Cognitive behavioral therapy (CBT) (*N* = 12–27.90%) (24–27, 29–32, 34, 41, 44, 45), CBT combined with another intervention (*N* = 2–4.65%) (31, 58), Psychoeducation (*N* = 13–30.23%) (28, 29, 33, 37, 41, 50, 51, 56, 57, 59–62), Neurocognitive (*N* = 5–11.62%) (34, 37, 40, 54), Narrative psychotherapy (*N* = 7–16.27%) (35, 38, 39, 46–48, 55), Yoga/Meditation (*N* = 4–10%) (23, 31, 42, 49). Table 2 shows the synthesis of the studies included in the systematic review.

**TABLE 2:** Synthesis of the studies included in the systematic review (*n* = 26).

Country (References)	N (%)	Study design	N (%)	Mental health conditions	N (%)	Primary outcomes	N (%)	Types of intervention	N (%)	Config- uration	N (%)	Intervention comparator	N (%)	Deliverer	N (%)
Bosnia (33, 38)	2 (5.13)	RCT	36 (92.30)	ADHD	7 (17.94)	Psychiatric symptoms	38 (97.43)	Cognitive behavioural therapy (CBT)	12 (27.90)	Individual	11 (25.58)	No intervention	32 (82.05)	Psychologist	14 (35.89)
Brazil (42, 49)	2 (5.13)	Non-randomised trial	2 (0.05)	PTSD	14 (35.90)	Emotional problems	1 (2.57)	CBT Plus another intervention	2 (4.65)	Group	21 (48.83)	Psychoeducation	7 (17.95)	Psychiatrist	5 (12.82)
Burundi (46)	1 (2.56)	Quasi-experimental	1 (0.02)	Anxiety	7 (17.94)			Narrative psychotherapy	7 (16.27)	Individual + group	10 (23.27)			Non-specialist professional	11 (28.20)
Chile (24, 32)	2 (5.13)			Depression	10 (25.64)			Psychoeducation	13 (30.23)	Not reported	1 (2.32)			Teacher	4 (10.25)
China (37, 53)	3 (7.69)							Neurocognitive	5 (11.62)					Lay person	4 (10.25)
Democratic. Republic Congo (41)	1 (2.56)			Conduct disorder	1 (2.48)			Yoga/Meditation/ Exercise	4 (9.30)					Not reported/not specified	1 (2.56)
Kenya (51, 56, 57, 59)	4 (10.26)														
India (45)	1 (2.56)														
Indonesia (48)	1 (2.56)														
Iran (23, 30, 31, 34, 36, 39, 40, 54)	8 (20.52)														
Lebanon (62)	1 (2.56)														
Malaysia (44, 60)	2 (5.13)														
Mexico (61)	1 (2.56)														
Nepal (35)	1 (2.56)														
Nigeria (27, 58)	2 (5.13)														
Palestine (26, 43)	2 (5.13)														
South Africa (55)	1 (2.56)														
Sri Lanka (28, 47)	2 (5.13)														
Turkey (25)	1 (2.56)														
Uganda (50)	1 (2.56)														

ADHD, attention deficit hyperactivity disorder; PTSD, post-traumatic stress disorder.

Table 3 shows the synthesis of the studies included in the systematic review and the risk of bias. There were 7 studies (17.94%) on ADHD (23, 36, 37, 39, 42, 53, 61), 14 studies (35.90%) on PTSD (28, 29, 33–35, 38, 41, 43, 46–48, 55, 57), 10 studies (25.64%) on depression (24, 27, 30, 32, 44, 45, 49, 50, 54, 56), and 7 studies (17.94%) on anxiety (25, 30, 31, 51, 59, 60, 62).

**TABLE 3:** Summary effect of interventions as compared to control/no intervention, and risk of bias classification by conditions and types of intervention.

		Effect interventions vs. comparators				Risk of bias		
		Positive	Negative	Neutral	Mixed	Low ROB	Some concerns	High ROB
	N (%)	N (%)	N (%)	N (%)	N (%)	N (%)	N (%)	N (%)
**Conditions**								
ADHD	7 (17.94)	5 (71.42)	0	2 (28.58)	0	1 (14.20)	3 (42.85)	3 (42.85)
PTSD	14 (35.90)	9 (64.28)	0	5 (35.72)	0	2 (14.28)	12 (85.72)	0
Anxiety	7 (17.94)	2 (28.57)	0	5 (71.43)	0	3 (42.85)	0	4 (57.15)
Depression	10 (25.64)	6 (60)	0	4 (40)	0	3 (30)	0	7 (70)
Conduct disorder	1 (2.48)	1 (100)				1 (100)		
Total	39	23 (58.97)	0	16 (41.03)	0	10 (7.69)	15 (53.84)	13 (38.47)
**Interventions**								
Cognitive behavioural therapy (CBT)	12 (27.90)*	7 (60.0)	0	5 (40.0)	0	1 (8.3)	7 (58.33)	4 (33.3)
CBT Plus another intervention	2 (4.65)	1 (50%)0	0	1 (50%)	0	1 (50)	1 (50)	0
Narrative psychotherapy	7 (16.27)	3 (33.34)	0	4 (66.66)	0	1 (14.29)	0	6 (85.71)
Psychoeducation	13 (30.23)*	9 (50)	0	4 (50)	0	6 (46.15)	2 (15.38)	5 (38.46)
Neurocognitive	5 (11.62)	3 (33.34)	0	2 (66.66)	0	2 (40)	1 (20)	2 (40)
Yoga/Meditation/Exercise	4 (9.30)	3 (100)	0	10	0	0	1 (25)	3 (75)
Total	43	16 (52.33)	0	14 (47.67)	0	11 (25.58)	12 (27.90)	20 (46.51)

*Some studies had more than one intervention group.

Eleven studies presented low risk of bias. A total of 12 studies had some concerns and 20 studies had high risk of bias.

Cognitive behavioral therapy was used to treat all conditions, but ADHD (24–27, 29, 30, 32, 34, 41, 43–45) and CD (58). CBT Plus another intervention was only investigated for anxiety and CD (31, 58). Narrative psychotherapy were used in seven studies; ADHD and PTSD (35, 37, 38, 46–48, 55). Psychoeducation were used in 13 studies (28, 29, 33, 37, 41, 50, 51, 56, 57, 59–62) for PTSD, ADHD, depression, and anxiety. Yoga/meditation/exercise were used in four studies (23, 36, 42, 49) in ADHD and depressive symptoms. Neurocognitive therapy was used in five studies (34, 37, 39, 40, 54) for ADHD, PTSD anxiety, and depression. Table 4 shows the interventions investigated per condition.

**TABLE 4:** Interventions investigated per condition.

Condition	Cognitive behavioural therapy (CBT)	CBT plus another intervention	Narrative psychotherapy	Psychoeducation	Neurocognitive	Yoga/Meditation/ Exercise
Anxiety	2 (16.66)	1 (50.00)	0	4 (30.76)	0	0
ADHD	0	0	1 (14.28)	2 (15.38)	2 (40.00)	3 (75.00)
Depression	5 (41.66)	0	0	2 (15.38)	2 (40.00)	1 (25.00)
PTSD	5 (41.66)	0	6 (85.72)	5 (38.46)	1 (20.00)	0
CD		1 (50.00)		0		
Total	12 (27.90)	2 (4.65)	7 (16.27)	13 (30.23)	5 (11.62)	4 (9.30)

ADHD, attention deficit hyperactivity disorder; PTSD, post-traumatic stress disorder; CD, conduct disorder.

### Pairwise comparisons

**FIGURE 2:**
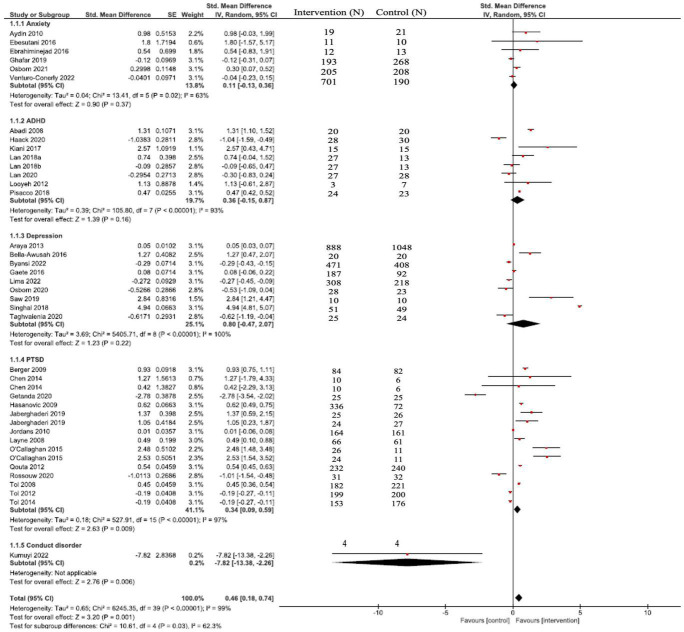
Forest plot comparing interventions with control.

In the 39 studies included in our review, we identified 43 different pairwise comparisons. We grouped interventions according to five types of mental health conditions based on the stated target of the intervention. Figure 2 show pooled SMD for all conditions, only PTSD (SMD = 0.61; 95% CI = 0.37–0.86) were statistically significant. Overall, interventions were superior to comparators SMD = 0.46 (0.18–0.74); not statistically significant for anxiety (SMD = 0.11; 95% CI = −0.13 to 0.36), ADHD (SMD = 0.36; 95% CI = −0.15 to 0.87), and for depression (SMD = 0.80; 95% CI = −0.47 to 2.07). For CD the sample size was very small, so the results are imprecise.

## Discussion

### Main results

In our review, we found 39 studies which assessed the efficacy/effectiveness of interventions for 6–18 years old child/adolescent to treat mental health problems in schools in LMICs. When stratified by conditions, pooled effects of interventions to treat anxiety, ADHD, depression were non-superior to comparators. For PTSD, significant effect was found. When SMD of all studies were pooled together, a significant effect was found, suggesting that, overall, interventions delivered in the school environment are effective in reducing mental health problems among children and adolescents.

### Comparison to other reviews

Other systematic reviews published in the literature are mostly focussed on universal interventions on mental health disease prevention and wellness promotion (13, 15, 16), with conflicting results–some reviews have found, for example small to medium STD to prevent depression and anxiety symptoms (13, 14) and to promote wellbeing (16). In other review, however, found no evidence of effects on preventing depression, anxiety, and PTSD (16).

Anxiety and depression are being reported together in school-based mental health literature synthesis (14, 63, 64). However, as showed in our results, school-based interventions reach different results for each condition, thus requiring future studies to deal separately considering universal or target programs (65).

Regarding PTSD and childhood trauma there is a growing body of evidence from systematic reviews associating the importance of cognitive behavioural therapies for reducing the risk of psychotic symptoms, improve well-being (66, 67) which is in agreement with our interventions effectiveness found in the literature.

Regarding ADHD, multiple psychosocial have been developed and empirically tested to improve ADHD symptoms, according to CADDRA Guidelines Work GROUP the evidence supports Cognitive Behavioural Therapy and Caregiver interventions. Other interventions, such as Physical Exercise and Mind–Body, still lack strong evidence to be supported (68). Our review found that multiple school-based psychosocial interventions have been tested in LMIC and single studies shows promising effects.

Offering interventions to prevent mental disorders and to promote mental health in schools should[could] contribute to improve access to care among young people with mental health problems by adopting task-share approaches that propose that mild and moderate mental disorders can be treated in the community by no-specialist professionals. Therefore, identifying treatment programmes (universal or targeted) that are effective when delivered in schools is key for the scaling up of mental health care. Our review contributes to the literature by identifying interventions that have been delivered in schools and proven effective.

### Additional reflection points

Most children and adolescents with mental health conditions do not receive evidence-based care or they are underdiagnosed, leading to chronicity of mental health symptoms and increased costs of care (69). The body of evidence applied in LMICS comes from HICs countries, however, different modes of living, social, cultural, and health system factors limit the generalisability and applicability of indirect evidence (70).

In our review, interventions were delivered by different professionals, for instance: teachers, researchers, community health workers, non-specialised health professionals, lay person. These professionals are well-recognised for educating and mobilising the community to increase demand for care (71). This is a valid strategy, and is in agreement with the literature (72–74), which suggests young people with mild and moderate symptoms can be treat in the community, and only more severe cases, which requires a greater level of care should be referral to specialised providers (75). Our results show that there is a potential for scaling up interventions to treat mental health problems in schools, helping to increase young people’s access to care in settings where health care professionals are scarce.

### Quality of the evidence

GRADE approach assess five factors: risk of bias, inconsistency, indirectness, imprecision, and publication bias and factors that increase the quality of evidence (large magnitude of an effect, dose-response gradient, effect of plausible residual confounding). Thus, as seen in Table 1: The SMD varied across studies and group of conditions/interventions, we believe the intervention protocols being studied, many different conditions, and different stage of disease were the cause of serious downgraded (−1), due to inconsistency among studies and imprecision of estimates. Additionally, another serious downgrade (−1), the study limitations (risk of bias).

Considering the broad evidence synthesised for interventions and conditions, we would make weak recommendation/very low-quality evidence, which means caregivers will need to allocate more time to shared decision making including individual patient’s circumstances, preferences, and values.

### Study limitations

The studies have limitations that should be highlighted. First, there are three main potential bias in the review process concerns: (1-) lack of reporting to allow us make judgement in the “Risk of bias 2.0” assessment; (2-) many conditions and many interventions were found, which increased clinical variability across the review, turning it in a more descriptive synthesis of the literature; (3-) lack of consistency of interventions protocols. Additionally, most trials were not registered, presenting another potential source of bias.

This review does not address comparison between effectiveness of interventions delivered in the school setting and other settings. It does show, however, that some interventions are effective for certain type of symptomatology. Once there is now evidence of interventions that could effectively treat mental health problems in school settings, additional studies should explore the feasibility of scalling up such intervention in the school system in different contexts and identify which factors would facilitate implementation in real world circumstances.

### Implications and recommendations for future researchers

Implementation of school-based interventions is conditioned on school attendance of children and adolescents which can vary among and within LMICs (9). Additionally, other factors can influence engagement and delivery, as this may require appropriate training for teachers and other school personnel, impact on their routine and require additional supportive, such as supervision, as well as additional material/economic resources (76, 77). Thus, planning of mental health programs are complex and need to consider the factors raised above.

Low- and middle-income countries are diverse/heterogenous countries. Yet, most of the evidence in the review comes from Iran, in the Western Asia region, and may not be easily transferrable to other LMIC due to cultural, economic, and other contextual differences.

The methodological quality of studies called our attention, we found a considerable number of studies with methodological concerns and high risk of bias.

Future studies should examine and describe in more detail the effectiveness of intervention’s components, such as frequency, delivery methods, etc., as well as implementation aspects that could guide policy and decision making for better mental health care.

## Conclusion

We presented a systematic review of school-based interventions for mental health problems in young people living in low- and middle-income countries. The evidence presented here is motivated by the uniqueness of school environment for such interventions, the fact that most children and adolescents in the world live in a LMIC context and no evidence synthesis have been previously organised.

The results indicated that school-based interventions for anxiety, PTSD, and ADHD in children and adolescents tested in LMICs showed a significant reduction of symptoms. The list of interventions from primary studies were: CBT was used to treat PTSD, anxiety, depression and CD. CBT Plus another intervention was only investigated for anxiety and CD. Narrative psychotherapies were used in ADHD and PTSD. Psychoeducation in PTSD, ADHD, depression, and anxiety. Yoga/meditation/exercise were used in ADHD and depressive symptoms. Neurocognitive therapy was used in ADHD, PTSD, anxiety, and depression.

## Author contributions

AG conceived and designed the study, contributed to the definition of the search strategy, and wrote the first version of the manuscript. WR and MH conceived and designed the study and contributed to the definition of the search strategy and the writing up of the manuscript. CM and CT contributed to the study design and revision of the manuscript. DM and CZ contributed to the study design, definition of the search strategy, and revision of the manuscript. SE-L conceived and designed the study and contributed to the definition of the search strategy and revision of the manuscript. All authors contributed to the article and approved the submitted version.
